# PMJDM: a multi-task joint detection model for plant disease identification

**DOI:** 10.3389/fpls.2025.1599671

**Published:** 2025-05-22

**Authors:** Rui Fu, Xuewei Wang, Shiyu Wang, Hao Sun

**Affiliations:** Shandong Province University Laboratory for Protected Horticulture, Weifang University of Science and Technology, Weifang, China

**Keywords:** plant disease detection, multi-task learning, candidate region generation, conditional random fields, dynamic weight adjustment

## Abstract

**Introduction:**

Plant disease detection is critical for ensuring agricultural productivity, yet traditional methods often suffer from inefficiencies and inaccuracies due to manual processes and limited adaptability.

**Methods:**

This paper presents the PlantDisease Multi-task Joint Detection Model (PMJDM), which integrates an enhanced ConvNeXt-based shared feature extraction, a texture-augmented N-RPN module with HOG/LBP metrics, multi-task branches for simultaneous plant species classification and disease detection, and CRF-based post-processing for spatial consistency. A dynamic weight adjustment mechanism is also employed to optimize task balance and improve robustness.

**Results:**

Evaluated on a 26,073-image dataset, PMJDM achieves 71.84% precision, 61.96% recall, and 61.83% mAP50, surpassing Faster - RCNN (51.49% mAP50) and YOLOv10x (59.52% mAP50) by 10.34% and 2.31%, respectively.

**Discussion:**

The superior performance of PMJDM is driven by multi-task synergy and texture - enhanced region proposals, offering an efficient solution for precision agriculture.

## 1 Introduction

**Figure 1:**
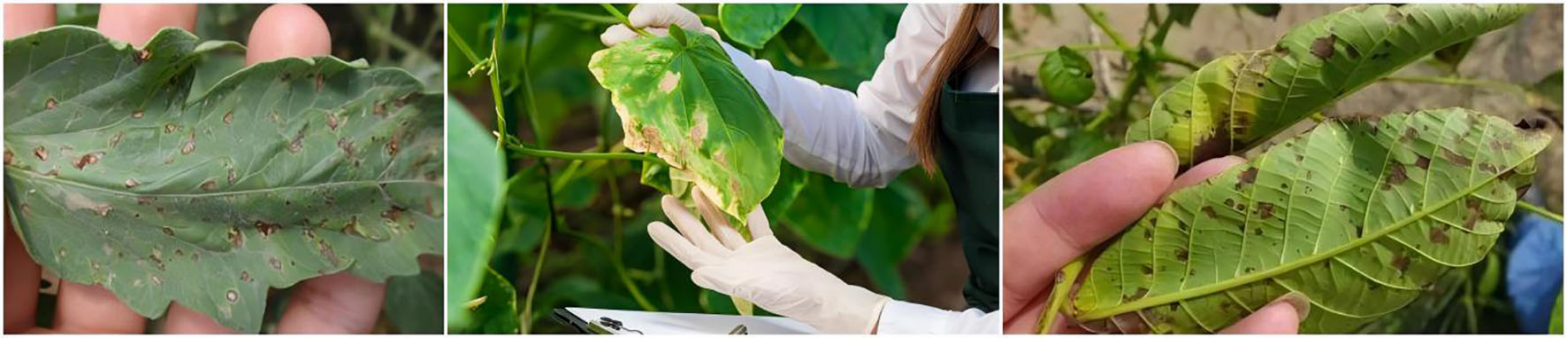
Detection of plant diseases using conventional manual methods.

Plant disease detection is a cornerstone of precision agriculture, significantly influencing crop yield, quality, and sustainability. With global food demand projected to increase by 70% by 2050, timely and accurate disease identification is critical to minimizing yield losses and reducing reliance on chemical pesticides. Traditional manual detection, which relies on expert visual inspection and laboratory analysis, is labor-intensive, subjective, and prone to errors under complex field conditions, as illustrated in **Figure 1**. Automated solutions based on computer vision and machine learning have emerged as scalable and efficient alternatives. However, these methods face significant challenges in real-world agricultural environments, highlighting the need for innovative approaches to enhance detection accuracy and robustness.

**Figure 2:**
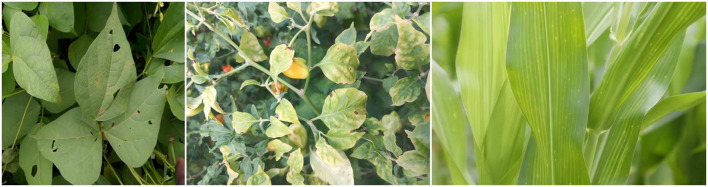
Diseased leaves of various plants, highlighting challenges in complex backgrounds and multiscale targets.

Despite advancements, key technical gaps remain in automated plant disease detection. First, multitask learning frameworks often face gradient conflicts between plant species classification and disease localization, leading to suboptimal model performance. Second, traditional region proposal methods like those in Faster-RCNN struggle to generate robust candidate regions in complex scenes with blurred leaf edges, multi-scale disease targets, or varying lighting conditions, as shown in **Figure 2**. Third, existing models are sensitive to local noise and texture variations, reducing classification and detection accuracy in diverse agricultural environments. These challenges drive the need for a unified model that can jointly optimize multiple tasks while maintaining robustness across varied conditions.

This study proposes the Plant Disease Multi-task Joint Detection Model (PMJDM), a novel framework addressing these technical gaps. PMJDM integrates: (1) a shared feature extraction module using an improved ConvNeXt backbone, (2) a texture-enhanced region proposal network (N-RPN) with HOG/LBP metrics, (3) multi-task branches for simultaneous classification and detection, and (4) conditional random field (CRF)-based post-processing for spatial consistency. A dynamic weight adjustment mechanism enables balanced multi-task optimization. Evaluation on a 26,073-image dataset shows PMJDM achieves 71.84% precision, 61.96% recall, and 61.83% mAP50, outperforming state-of-the-art models including Faster-RCNN and YOLOv10x. The key contributions are:

- Dual-Task Joint Inference Framework: We construct a shared feature extraction module based on an improved ConvNeXt network, combined with a dual-branch parallel structure. This framework enables simultaneous plant multi-label classification and disease target detection within a single model, significantly reducing computational redundancy;
- Dynamic Gradient Balancing Mechanism: An adaptive weight adjustment function based on loss ratios is proposed, which calculates loss differences between classification and detection tasks in real-time to dynamically optimize task weights, thereby alleviating multi-task conflicts;
- Texture-Enhanced Candidate Region Generation: The region proposal network (N-RPN) incorporates HOG/LBP texture similarity metrics with geometrically adaptive sampling strategies using deformable convolution, improving detection robustness for multi-scale targets in complex backgrounds;
- Context-Aware Post-Processing Optimization: A Conditional Random Field (CRF) correction module is designed for plant classification tasks, integrating spatial constraints with texture feature optimization to effectively suppress local noise interference;
- High-Efficiency Deployment Validation: The model achieves 61.83% mAP50 detection accuracy with 49.1M parameters through channel attention compression and multi-scale feature fusion, reaching an inference speed of 113 FPS.

## 2 Literature review

Plant disease detection has evolved significantly, transitioning from manual inspection to automated systems leveraging image processing, machine learning, and deep learning. This section reviews key developments, categorizing them into traditional methods, machine learning approaches, deep learning models, and multi-task learning frameworks, while highlighting their limitations and the gaps addressed by PMJDM.

### 2.1 Traditional methods

Early plant disease detection relied on expert manual inspection through visual symptom analysis and laboratory tests. While foundational, these methods are inefficient and subjective, as shown in **Figure 2**. Automated traditional approaches employed image processing techniques to extract color and texture features. For example, Loranger et al. (2024) developed a “Colour Analyzer” tool using HSV and Lab* color models to precisely measure leaf lesion areas, supporting plant immunity research. Similarly, Bhujade et al. (2024) proposed the OptCFA method combining GABF, AmPel, and E SGF for image denoising, outperforming conventional filters. Nevertheless, these methods remain limited in adapting to diverse plant species and complex backgrounds, restricting their practical application.

### 2.2 Machine learning approaches

Machine learning enabled automated feature extraction and classification, outperforming traditional methods in efficiency. Sahu and Pandey (2023) developed the HRF-MCSVM model incorporating spatial fuzzy C-means for segmentation and feature preprocessing, which improved leaf disease detection accuracy. Selvaraj and Ananth (2023) created a SqueezeNet model with RCSO for root disease classification, achieving high sensitivity under low-power constraints. Luaces et al. (2011) implemented an early warning system for coffee rust that reduced pesticide application. Xie et al. (2023) proposed a Salp Swarm-based feature selection method to enhance classification efficiency. While these approaches represent significant advances, machine learning models still face challenges in generalizing across diverse datasets and depend heavily on manual feature engineering, constraining their scalability.

### 2.3 Deep learning models

Deep learning has revolutionized plant disease detection by enabling end-to-end feature learning. Singletask models like Faster-RCNN (Ren et al., 2015) and YOLO variants (Huang et al., 2023) excel in detection but lack integrated classification capabilities. For example, Li et al. (2025) proposed APNet, achieving 87.1% accuracy on apricot tree diseases using an adaptive thresholding module. Deng et al. (2023) introduced MC-UNet, a lightweight segmentation model with 91.32% accuracy on tomato leaf diseases. Shafik et al. (2024) developed PDDNet models, achieving up to 97.79% accuracy on the PlantVillage dataset. Singh et al. (2024) combined LeafyGAN and MobileViT, reaching 99.92% accuracy on PlantVillage. However, these models often focus on single tasks, facing challenges in multi-task integration and computational efficiency in resource-constrained settings. In a related application, Vaghela et al. (2025) demonstrated YOLO V8’s effectiveness in land cover classification for agricultural field identification, achieving high accuracy with different model variants.

### 2.4 Multi-task and lightweight models

Multi-task learning addresses the need for simultaneous classification and detection. Lin et al. (2024) proposed a dual-branch network combining CNN and Vision Transformer features, achieving 88.74% accuracy on the AI Challenger 2018 dataset. Li and Shen (2024) introduced DiCaN, enhancing performance through wavelet decomposition and cross-frequency attention. Wu et al. (2024) fused Transformer and CNN features for marine red tide classification, achieving 87% accuracy. Lightweight models, such as Huang et al. (2023)’s YOLOR-based student models (60.4% mAP@.5) and Zhang et al. (2024)’s U-shaped Transformer, prioritize efficiency but compromise accuracy in complex scenes. Nawaz et al. (2024) proposed CoffeeNet, achieving 98.54% accuracy with an improved CenterNet, though its single-task focus limits applicability. Similarly, Srinivasu et al. (2024) developed an XAI-driven crop recommendation system using neural networks and optimization techniques, demonstrating AI’s potential in precision agriculture.

### 2.5 Limitations and gaps

Existing methods face several limitations. Single-task deep learning models cannot jointly optimize classification and detection, resulting in computational redundancy. Multi-task models experience gradient conflicts that lead to unbalanced optimization. Traditional region proposal networks, such as those in FasterRCNN, demonstrate poor robustness in complex agricultural scenes with multi-scale targets or illumination variations. Additionally, their sensitivity to local noise and texture changes degrades performance. PMJDM addresses these gaps through: (1) a dual-task joint framework, (2) texture-enhanced region proposals with HOG/LBP metrics, (3) dynamic weight balancing, and (4) CRF-based post-processing, achieving superior accuracy and efficiency for real-world plant disease detection.

## 3 Method

**Figure 3:**
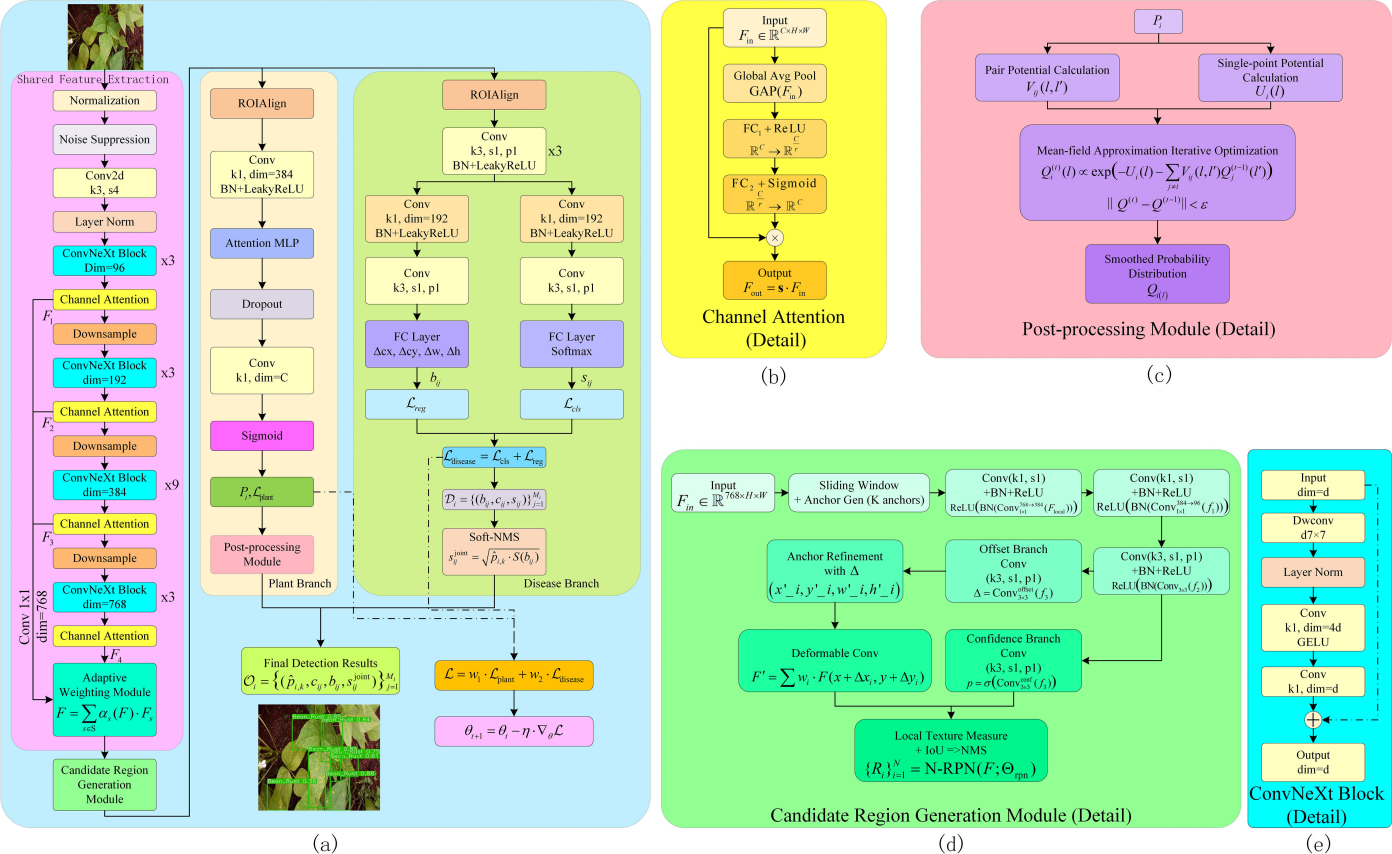
Complete structure diagram of PMJDM. **(a)** shows the overall architecture: input images first pass through the shared feature extraction module to generate feature map *F*, which is processed by the candidate region generation module. The features then feed into parallel plant classification and disease detection branches to produce respective outputs. Finally, results from both branches are integrated to generate the final output, with total loss updated via dynamic weight adjustment. **(b)** details the channel attention module, producing weighted feature map *F_out_* through global average pooling, fully connected layers, and Sigmoid activation. **(c)** illustrates the post-processing module, containing pairwise potential calculation, unary potential calculation, and mean-field approximation iterative optimization, outputting smoothed probability distribution *Q*_(_ *_i,l_*_)_. **(d)** presents the candidate region generation module, including sliding window, anchor refinement, deformable convolution, confidence branch, and local texture measurement to generate candidate regions. **(e)** details the ConvNeXt Block structure, comprising 7×7 depth-wise convolution, layer normalization (LN), 1×1 convolutions with channel expansion/compression, GELU activation, and residual connections.

This study proposes the Plant Disease Multi-task Joint Detection Model (PMJDM), a novel framework designed for simultaneous plant species classification and disease localization in complex agricultural environments, as shown in **Figure 3a**. PMJDM addresses key challenges in plant disease detection, including multi-task gradient conflicts, poor robustness in diverse backgrounds, and sensitivity to local noise. The model processes 640×640 input images with initial preprocessing for normalization and noise reduction. A shared feature extraction module based on an improved ConvNeXt backbone produces multi-scale semantic features. An adaptive candidate region generation module (N-RPN) subsequently identifies potential disease regions. Multi-task branches execute parallel plant classification and disease detection, followed by conditional random field (CRF)-based post-processing to improve spatial consistency. A dynamic weight adjustment mechanism balances task losses during training to ensure coordinated optimization. Soft non-maximum suppression (NMS) refines detection outputs, while end-to-end backpropagation optimizes the model. Below, we detail each module, emphasizing technical design, theoretical rationale, and their role in overcoming specific challenges.

### 3.1 Shared feature extraction

In the shared feature extraction module, this method starts with the input image *I* (640×640×3), first passing it through a preprocessing layer for normalization and noise suppression. Then, an improved ConvNeXt backbone network is used to extract features from the image. The improvement lies in embedding a multi-scale adaptive attention mechanism into the traditional ConvNeXt network (Liu et al., 2022), which can automatically adjust the convolution kernel parameters based on local feature statistics. Additionally, an extra channel attention module is introduced in the intermediate layers to enhance local details and edge features, as shown in **Figure 3b**. Specifically, after initial dynamic convolution processing, the image data enters the ConvNeXt backbone network, where it undergoes successive convolution, normalization, activation, and downsampling operations to obtain the multi-channel feature map *F* ∈ ℝ*^H/^* ^16×^ *^W/^* ^16×768^. The backbone begins with a stem layer using a 7×7 convolution with stride 2 for initial downsampling, followed by multiple ConvNeXt Blocks with stride 1 to preserve spatial resolution in deeper layers. Each ConvNeXt Block, as shown in **Figure 3e**, follows the standard design (Liu et al., 2022). It starts with a 7×7 depth-wise convolution (maintaining the input channel dimension), followed by layer normalization (LN), a 1×1 point-wise convolution expanding to a larger channel dimension, a GELU activation, and another 1×1 point-wise convolution compressing back to the original channel dimension. A residual connection adds the input to the output, ensuring training stability. In the final stage, the backbone outputs 768 channels, achieved through a series of Blocks with progressively increasing channel dimensions, following the standard ConvNeXt-Tiny configuration.During this process, a set of parameterized adaptive weighting functions *A*(·) is introduced within the network, which fuses features from different scales using local gradients and statistical information, ultimately yielding the final enhanced feature representation. As shown in Equation 1:


(1) F=A(ℬ(I)),

Where ℬ(·) represents the convolution operation process of the traditional ConvNeXt network. Specifically, the computation of the adaptive weighting function A(·) can be expressed as Equation 2:


(2) $$A\left(F\right)=\sum_{s\in S}\alpha_{s}\left(F\right)\cdot F_{s},$$

Where S denotes the set of features at different scales, *F_s_* is the feature map at scale *s*, and $\alpha_{s}\left(F\right)$ is the adaptive weight coefficient related to scale *s*, dynamically generated from the local gradient information and inter-channel statistical distribution of the feature map *F*. As shown in Equation 3:


(3) $$\alpha_{s}\left(F\right)=\sigma \left(W_{s}\cdot \text{GAP}\left(F\right)+b_{s}\right),$$

Where **W** *_s_* and *b_s_* are learnable parameters, GAP(·) represents the global average pooling operation, and σ(·) is the Sigmoid activation function. This design effectively enhances the feature responses of plant leaf edges and disease spots, thereby providing more refined semantic and local information for subsequent candidate region generation.

### 3.2 Candidate region generation

The candidate region generation module, termed N-RPN, efficiently identifies potential disease regions on the shared feature map *F*, as shown in **Figure 3d**. Traditional region proposal networks (e.g., in Faster-RCNN) rely solely on intersection-over-union (IoU) metrics, often failing in scenes with overlapping or multi-scale targets. N-RPN addresses this by integrating Histogram of Oriented Gradients (HOG) and Local Binary Patterns (LBP) for texture similarity, selected for their robustness to illumination changes and capacity to capture edge orientations (HOG) and local texture patterns (LBP). These metrics complement IoU, improving region quality in complex backgrounds.

The module employs a sliding window strategy to generate *K* = 9 anchor boxes per location, combining three scales (8, 16, 32) and three aspect ratios (0.5, 1, 2). A lightweight feedforward network refines anchors using 1×1 convolutions for channel reduction (768 to 384, then 384 to 96) and a 3×3 convolution for spatial feature extraction, all with stride 1 to preserve resolution. As shown in Equations 4–6:


(4) $$f_{1}=\text{ReLU }(\text{BN }({\text{Conv}}_{1\times 1}^{768\to 384}\left(F_{\text{local}}\right))),\;f_{1}\in {\mathbb{R}}^{H\times W\times 384},$$

(5) $$f_{2}=\text{ReLU }(\text{BN }({\text{Conv}}_{1\times 1}^{384\to 96}\left(f_{1}\right))),\;f_{2}\in {\mathbb{R}}^{H\times W\times 96},$$

(6) $$f_{3}=\text{ReLU }(\text{BN }(\text{Conv}3\times 3(f_{2}))),\;f_{3}\in {\mathbb{R}}^{H\times W\times 96}.$$

An offset branch predicts bounding box adjustments. As shown in Equation 7:


(7) $$\text{Δ}={\text{Conv}}_{3\times 3}^{\text{offset}}\left(f_{3}\right),\text{ Δ}\in {\mathbb{R}}^{H\times W\times 4K},$$

where Δ=(Δx,Δy,Δw,Δh) adjusts anchor coordinates and scales. A confidence branch predicts region scores. As shown in Equation 8:


(8) $$p=\sigma \left({\text{Conv}}_{3\times 3}^{\text{conf}}\left(f_{3}\right)\right),\;p\in {\mathbb{R}}^{H\times W\times K}.$$

Deformable convolution further adapts feature sampling to irregular disease shapes. As shown in Equation 9:


(9) $$F^{\prime }=\sum_{i}w_{i}\cdot F\left(x+\text{Δ}x_{i},y+\text{Δ}y_{i}\right),$$

where *w_i_* are learnable weights. An adaptive suppression mechanism combines IoU and texture similarity. As shown in Equation 10:


(10) $$S\left(R_{i},R_{j}\right)=\lambda_{1}\cdot \text{IoU }(R_{i},R_{j})+\lambda_{2}\cdot \text{TextureSim }(R_{i},R_{j}),$$

with *λ*_1_ = 0.7 and *λ*_2_ = 0.3, ensuring robust region selection. The final candidate regions as shown in Equation 11:


(11) $${\left\{R_{i}\right\}}_{i=1}^{N}=\text{N}-\text{RPN }(F;{\text{Θ}}_{\text{rpn}}).$$

This design enhances detection of multi-scale and overlapping disease targets, critical for agricultural scenes.

### 3.3 Multi-task branches

The multi-task branches process fixed-size features *F_i_* (7×7×256) extracted via ROIAlign from each candidate region *R_i_*, enabling simultaneous plant species classification and disease detection. The plant classification branch uses a multi-label approach, as a single region may contain multiple species (e.g., mixed crops). Features are processed through a 1×1 convolution (384 channels), batch normalization, and ReLU, followed by a multi-layer perceptron (MLP) with attention weights to enhance semantic representation. The Sigmoid activation ensures multi-label probabilities. As shown in Equation 12:


(12) $$P_{i}=\text{Sigmoid }(W_{2}*\text{Dropout }(\text{ReLU }(\text{BN }(W_{1}*F_{i}+b_{1})))+b_{2}),$$

with loss computed via binary cross-entropy. As shown in Equation 13:


(13) $${ℒ}_{\text{plant}}=-\frac{1}{C}\sum_{c=1}^{C}[y_{i,c}\text{log }(p_{i,c})+\left(1-y_{i,c}\right)\text{log }(1-p_{i,c})].$$

Sigmoid is chosen over Softmax to handle non-exclusive labels, addressing the multi-label nature of plant classification.

The disease detection branch employs a feature encoder with convolutional layers, batch normalization, and LeakyReLU (slope 0.1) to capture local disease patterns. It splits into regression and classification sub-branches. The regression sub-branch predicts bounding box coordinates using L1 and generalized IoU (GIoU) losses. As shown in Equation 14:


(14) $${ℒ}_{\text{reg}}=\frac{1}{M_{i}}\sum_{j=1}^{M_{i}}[\alpha \cdot \|b_{ij}-b_{ij}^{*}{\|}_{1}+\beta \cdot \left(1-\text{GIoU }(b_{ij},b_{ij}^{*}\right))],$$

with α=0.5 , β=1.0 . The classification sub-branch uses focal loss to address class imbalance. As shown in Equation 15:


(15) $${ℒ}_{\text{cls}}=-\frac{1}{M_{i}}\sum_{j=1}^{M_{i}}[\left(1-s_{ij}{)}^{\gamma }\text{log }(s_{ij}\right)],$$

with *γ* = 2.0. The total disease detection loss is. As shown in Equation 16:


(16) $${ℒ}_{\text{disease}}={ℒ}_{\text{cls}}+{ℒ}_{\text{reg}}.$$

Focal loss prioritizes hard-to-classify disease instances, improving detection in cluttered scenes. The branch outputs. As shown in Equation 17:


(17) $$D_{i}={\left\{\left(b_{ij},c_{ij},s_{ij}\right)\right\}}_{j=1}^{M_{i}}.$$

This dual-branch design minimizes computational redundancy while addressing the distinct requirements of classification and detection.

### 3.4 Post-processing of plant category branch - conditional random field

The plant classification branch’s preliminary predictions P*_i_* may suffer from local noise due to uneven illumination or texture variations. Since plant regions exhibit spatial continuity, we employ a CRF-based post-processing module, as shown in **Figure 3c**, to enforce spatial consistency. CRF models the probability distribution over plant labels by combining unary and pairwise potentials. The unary potential measures the cost of assigning label *l* to a pixel or sub-region. As shown in Equation 18:


(18) $$U_{i}\left(l\right)=-\text{log }{\hat{p}}_{i,l},$$

where ${\hat{p}}_{i,l}$ is the preliminary probability for label *l*. The pairwise potential incorporates spatial and texture constraints. As shown in Equation 19:


(19) $$V_{ij}\left(l,l^{\prime }\right)=\mu \left(l,l^{\prime }\right)\cdot \text{exp }(-\frac{\|p_{i}-p_{j}{\|}^{2}}{2\sigma_{\alpha }^{2}}-\frac{\|I_{i}-I_{j}{\|}^{2}}{2\sigma_{\beta }^{2}}),$$

where $\mu \left(l,l^{\prime }\right)={⊮}_{l\neq l^{\prime }},p_{i},p_{j}$ are spatial coordinates, $I_{i},I_{j}$ are texture features, and $\sigma_{\alpha }=5$ , $\sigma_{\beta }=3$ control spatial and texture influence. Mean-field approximation iteratively updates the smoothed probability. As shown in Equation 20:


(20) $$Q_{i}\left(l\right)\propto \text{exp }(-U_{i}\left(l\right)-\sum_{j\neq i}V_{ij}\left(l,l^{\prime }\right)Q_{j}\left(l^{\prime }\right)).$$

The Conditional Random Field (CRF) is adopted for its capacity to model pixel-level dependencies, effectively mitigating misclassifications induced by local noise (e.g., shadows) while maintaining precise plant region boundaries—a capability absent in conventional smoothing filters.

### 3.5 Dynamic weight adjustment

Multi-task training risks domination by a single task due to differing loss scales. To address this, we propose a dynamic weight adjustment mechanism based on the loss ratio between L_plant_ and L_disease_. As shown in Equations 21, 22:


(21) $${ℒ}_{\text{plant}}=-\frac{1}{C}\sum_{c=1}^{C}[y_{i,c}\text{log }(p_{i,c})+\left(1-y_{i,c}\right)\text{log }(1-p_{i,c})],$$

(22) $${ℒ}_{\text{disease}}=\frac{1}{M_{i}}\sum_{j=1}^{M_{i}}[-\left(1-s_{ij}{)}^{\gamma }\text{log }(s_{ij}\right)+\alpha \|b_{ij}-b_{ij}^{*}{\|}_{1}+\beta \left(1-\text{GIoU }(b_{ij},b_{ij}^{*}\right))].$$

The loss ratio as shown in Equation 23:


(23) $$\delta =\frac{{ℒ}_{\text{plant}}-{ℒ}_{\text{disease}}}{{ℒ}_{\mathrm{plant}}+{ℒ}_{\mathrm{disease}}+\epsilon },$$

with $\epsilon =10^{-6}$ . Task weights are updated using the hyperbolic tangent function for smooth transitions. As shown in Equation 24:


(24) $$w_{1}=\frac{1-\text{tanh}\;(\delta )}{2},\;w_{2}=\frac{1+\text{tanh}\;(\delta )}{2}.$$

The total loss as shown in Equation 25:


(25) $$ℒ=w_{1}\cdot {ℒ}_{\text{plant}}+w_{2}\cdot {ℒ}_{\text{disease}}.$$

The tanh function ensures gradual weight adjustments, preventing abrupt shifts that could destabilize training. This mechanism balances gradient contributions, enabling stable convergence across tasks.

### 3.6 Final output and model update

In inference, each candidate region *R_i_* outputs a CRF-corrected plant category probability ${\hat{P}}_{i}$ and disease detection results *𝒟_i_*. Soft NMS adjusts detection confidences based on overlap. As shown in Equation 26:


(26) $$s_{ij}^{\text{joint}}=\sqrt{{\hat{p}}_{i,k}\cdot S\left(b_{ij}\right)},$$

where $S\left(b_{ij}\right)$ is the soft suppression factor. The joint output as shown in Equation 27:


(27) $$O_{i}={\left\{\left({\hat{p}}_{i,k},c_{ij},b_{ij},s_{ij}^{\text{joint}}\right)\right\}}_{j=1}^{M_{i}}.$$

Model parameters are updated via backpropagation. As shown in Equation 28:


(28) $$\theta_{t+1}=\theta_{t}-\eta \cdot \nabla_{\theta }ℒ,$$

with learning rate *η* = 0.002. This end-to-end framework, enhanced by dynamic weight adjustment, ensures robust joint detection and classification in complex agricultural scenarios.

## 4 Results

### 4.1 Dataset and experimental setup

#### 4.1.1 Dataset

**Figure 4:**
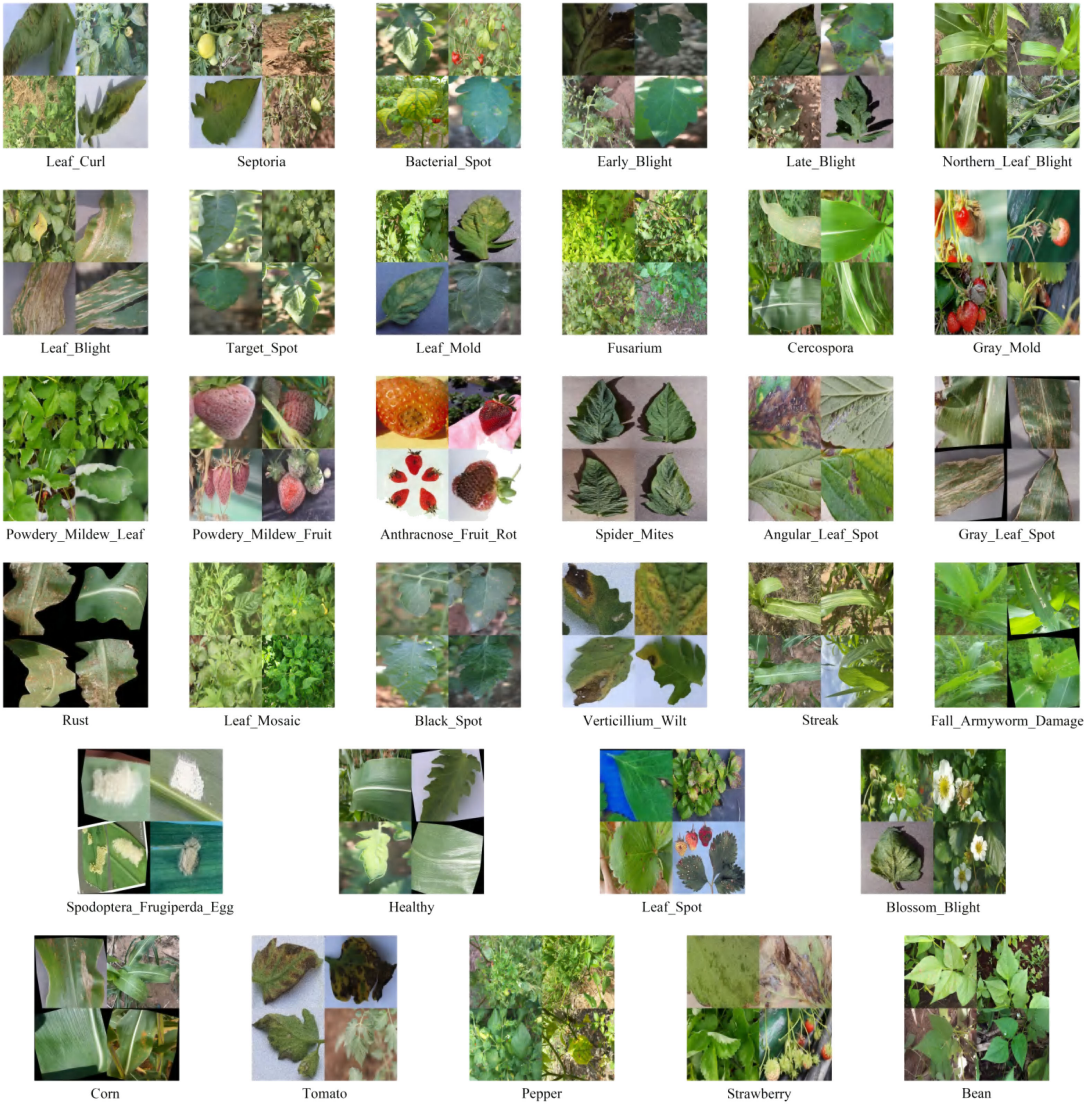
Dataset example. The first five rows are disease labels, and the last row is the plant label.

This study utilizes a dataset of 26,073 annotated images from multiple sources: 4,511 images from agricultural field collections, 8,339 from commercial datasets, and 13,223 from open-source repositories (links provided in the declaration). We standardize all images to 640×640 pixel resolution for deep learning compatibility. The annotation system includes two files per image: a plant species classification file (5 categories) and a disease detection file with bounding boxes (28 categories). We divide the dataset into training (70%, 18,251 images), validation (20%, 5,215 images), and testing (10%, 2,607 images) sets. **Figure 4** demonstrates representative samples across disease and plant categories, highlighting the dataset’s diversity.

#### 4.1.2 Implementation details

We develop and train the PMJDM model using PyTorch 2.2.2 with CUDA 12.1 on an NVIDIA GeForce RTX 4090 GPU. Our experimental platform consists of a Windows system with 64GB RAM to ensure efficient memory management during training. The 150-epoch training protocol employs a cosine learning rate scheduler that anneals the rate from 0.002 to 0.00002 for optimal convergence. The anchor configuration utilizes 9 anchors per location across three scales ([8, 16, 32]) and three aspect ratios ([0.5, 1, 2]), with a base size of 8 pixels. **Table 1** documents the core implementation parameters for reproducibility, while **Table 2** specifies the complete hyperparameter set.

**Table 1:** Implementation parameters for PMJDM training.

Parameter	Value
Framework	PyTorch 2.2.2
CUDA Version	12.1
GPU	NVIDIA GeForce RTX 4090
Operating System	Windows
RAM	64GB
Learning Rate Scheduler	Cosine

**Table 2:** Hyperparameters used in PMJDM training.

Hyperparameter	Value
Initial Learning Rate	0.002
Final Learning Rate	0.00002
Batch Size	16
Training Epochs	150
Optimizer	Adam
Anchor Number (K)	9
Anchor Base Size	8
Anchor Scales	[8, 16, 32]
Anchor Aspect Ratios	[0.5, 1, 2]
CRF *σ_α_*	5
CRF *σ_β_*	3
Dynamic Weight Adjustment *ϵ*	1e-6

In the experiment, precision, recall, mAP50, and mAP50–95 were used as evaluation metrics to assess the model’s performance.

### 4.2 Comparative experiment

As shown in **Table 3**, PMJDM exhibits clear superiority across key performance metrics: Precision, Recall, mAP50, and mAP50-95. Its precision reaches 71.84%, significantly outperforming Faster-RCNN’s 59.54% (a 12.3% gap), YOLOv8x’s 67.23% (4.61% lower), and YOLOv10x’s 67.53% (4.31% lower). This indicates PMJDM’s exceptional ability to reduce false positives, making it ideal for high-precision scenarios. Recall is at 61.96%, surpassing Mask R-CNN’s 52.27% by nearly 10%, YOLOv9e’s 55.97% by approximately 6%, and YOLOv10x’s 57.37% by 4.59%, demonstrating PMJDM’s reliability in detecting true positives and minimizing misses.

**Table 3:** Comparison of object detection model performance metrics.

Model	Precision	Recall	mAP50	mAP50-95
Faster-RCNN	59.54%	52.65%	51.49%	35.98%
Mask R-CNN	59.17%	52.27%	51.53%	36.02%
YOLOv8x	67.23%	55.49%	58.16%	40.57%
YOLOv9e	66.72%	55.97%	58.83%	40.82%
YOLOv10x	67.53%	57.37%	59.52%	41.37%
YOLO11x	66.81%	56.28%	59.06%	41.01%
PMJDM (our)	71.84%	61.96%	61.83%	42.69%

For mAP50, PMJDM achieves 61.83%, exceeding Faster-RCNN’s 51.49% (10.34% lower), YOLO11x’s 59.06% (2.77% lower), and YOLOv10x’s 59.52% (2.31% lower). This reflects superior average precision at an IoU threshold of 0.5, highlighting excellent localization and classification capabilities. Under the stricter mAP50–95 metric, PMJDM scores 42.69%, compared to YOLOv10x’s 41.37% (1.32% lower), YOLOv8x’s 40.57% (2.12% lower), and Faster-RCNN’s 31.62% (11.07% lower). This proves PMJDM’s consistent performance across varying IoU thresholds and its robustness under stringent localization requirements. Overall, PMJDM’s lead across all metrics underscores its efficiency and accuracy in complex detection scenarios, as visualized in **Table 3**, making it highly suitable for high-performance object detection applications.

**Figure 5:**
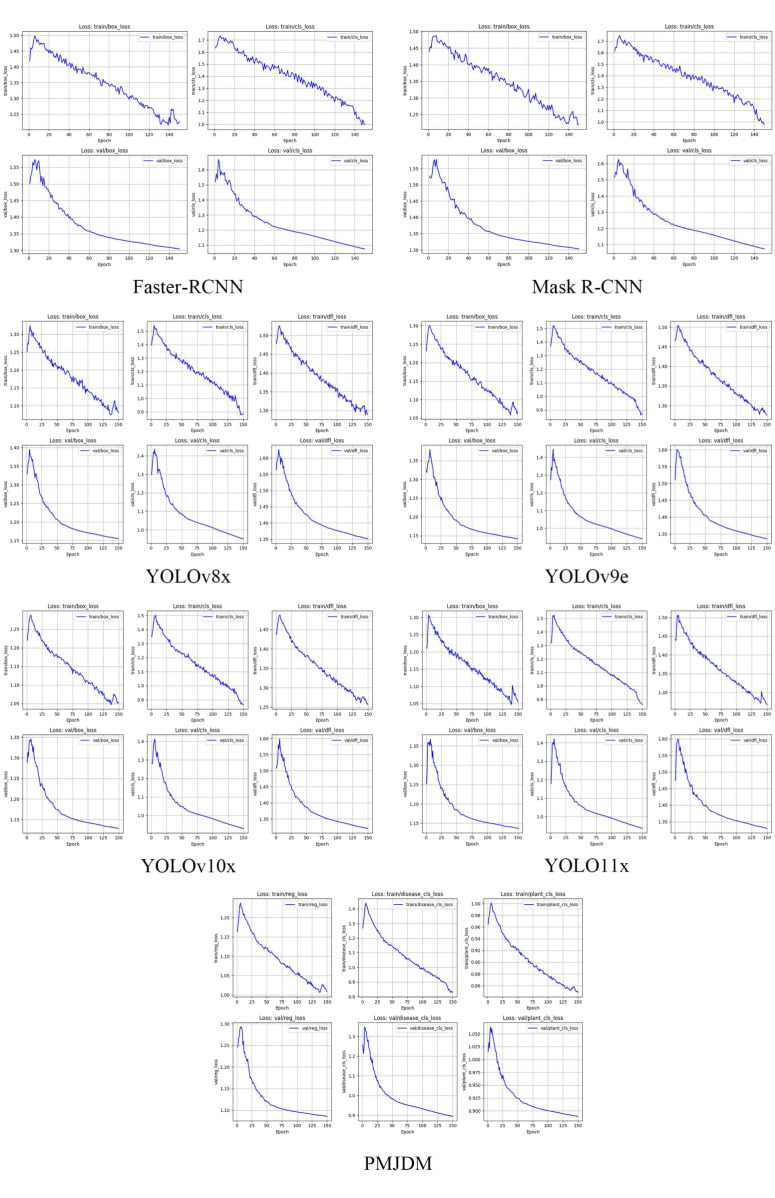
Training and validation loss curves for each model.

The loss curves in **Figure 5** illustrate the training and validation losses for several models, including Faster-RCNN, Mask R-CNN, YOLOv8x, YOLOv9e, YOLOv10x, YOLOv11x and PMJDM,. The PMJDM model stands out for its exceptional balance between disease detection and plant classification tasks. During training, PMJDM’s regression loss decreases from 1.1632 to 1.0087, disease classification loss from 1.2685 to 0.8310, and plant classification loss from 0.9648 to 0.8483, while validation losses show a regression loss drop from 1.2462 to 1.0861, disease classification loss from 1.2571 to 0.8949, and plant classification loss from 1.0151 to 0.8895. The disease and plant classification losses exhibit similar declining trends and converge to close final values, demonstrating PMJDM’s effective balance between these tasks, achieved through a dynamic weight adjustment mechanism that ensures equitable loss contributions. In contrast, Faster-RCNN and Mask R-CNN focus solely on bounding box and classification losses without multi-task balancing, and YOLO series models, while strong in box and classification losses, lack explicit task balance mechanisms. Furthermore, PMJDM’s training and validation losses remain closely aligned, indicating minimal overfitting and robust generalization capabilities.

**Figure 6:**
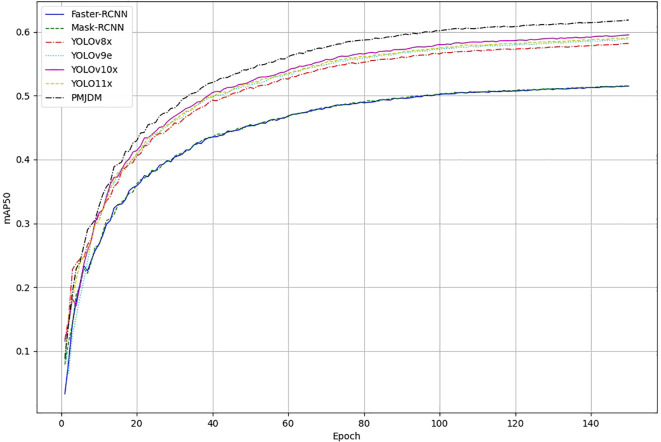
Training progression of object detection models: mean average precision (mAP50) Over 150 Epochs. The graph compares the performance of Faster-RCNN, Mask-RCNN, YOLOv8x, YOLOv9e, YOLOv10x, YOLO11x, and PMJDM, highlighting PMJDM’s consistent superiority throughout the training process.

The training progression of the evaluated object detection models, as depicted in **Figure 6**, further demonstrates PMJDM’s superiority. Over 140 epochs, all models initially exhibit a rapid increase in mAP50–95 within the first 40 epochs, followed by a performance divergence between epochs 40 and 100. During this phase, PMJDM and YOLO series models (YOLOv9e, YOLOv10x, YOLO11x) exceed 50%, while Faster-RCNN and Mask-RCNN stabilize around 40%-45%. By epoch 140, the mAP values plateau, with PMJDM achieving 61.83%, followed by YOLOv10x at 59.52% and YOLO11x at 59.05%. In contrast, Faster-RCNN and Mask-RCNN lag behind at 51.49% and 51.53%, respectively. This trend highlights PMJDM’s consistent lead throughout the training process, aligning with its top performance in **Table 3**, and establishes it as a robust choice for high-performance object detection tasks.

From **Table 4**, PMJDM, with 49.1M parameters, 145.76 GFLOPs, 113 FPS, and 61.83% mAP50, showcases remarkable advantages. It balances high accuracy with a lightweight design and efficient inference, ideal for embedded devices or real-time applications. In contrast, YOLOv8x has 68.2M parameters, 258.3 GFLOPs, 64 FPS, and 58.16% mAP50. PMJDM reduces parameters by 19.1M (28%), cuts computation by 112.54 GFLOPs (43.6%), increases speed by 49 FPS (76.6%), and improves mAP50 by 3.67%, demonstrating enhanced accuracy with lower resource demands, likely due to an optimized network structure.

**Table 4:** Efficiency and performance comparison of object detection models.

Model	Parameters (M)	GFLOPs	FPS	mAP50
Faster-RCNN	41.7	134.38	123	51.49%
Mask R-CNN	44.4	134.38	122	51.53%
YOLOv8x	68.2	258.3	64	58.16%
YOLOv9e	58.1	192.5	85	58.83%
YOLOv10x	31.7	171.4	96	59.52%
YOLO11x	56.9	195.7	84	59.06%
PMJDM (Our)	49.1	145.76	113	61.83%

Compared to YOLOv10x, with 31.7M parameters (17.4M fewer than PMJDM), 171.4 GFLOPs, 96 FPS, and 59.52% mAP50, PMJDM achieves a 2.31% higher mAP50 and 17 FPS faster speed, with 25.64 fewer GFLOPs. This suggests PMJDM trades a moderate increase in parameters for superior precision and efficiency, leveraging advanced computational techniques. Against heavier models like Faster-RCNN (137.1M parameters, 370.2 GFLOPs, 25 FPS, 51.49% mAP50), PMJDM’s advantages are even more pronounced. As illustrated in **Table 4**, its exceptional balance of efficiency and accuracy highlights its practicality for real-time, high-precision detection tasks.

**Figure 7:**
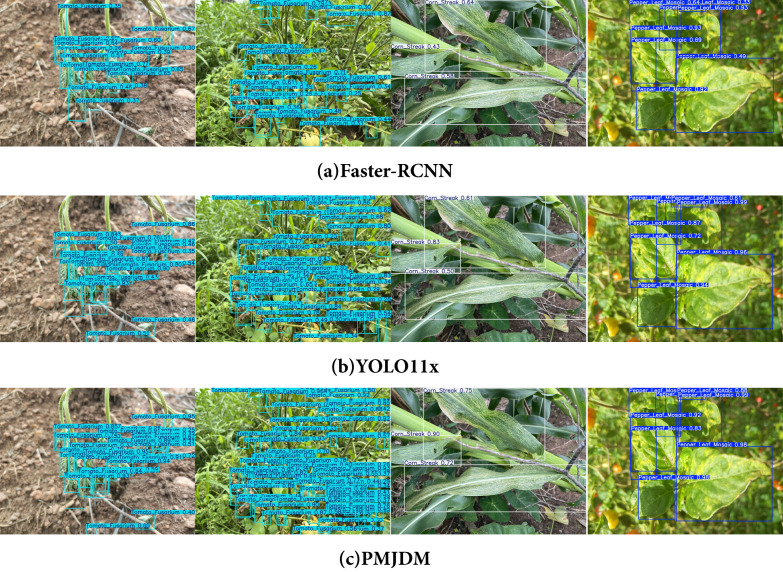
Comparison of the detection results of Faster-RCNN, YOLO11x, and PMJDM. **(a)** Faster-RCNN, **(b)** YOLOX11x, **(c)** PMJDM.

In **Figure 7**, the comparison of detection results among Faster-RCNN, YOLO11x, and PMJDM fully confirms the superiority of the proposed method. The first two sets of visualization results show that PMJDM generates more detection boxes than the comparison models, especially in complex scenes with blurred leaf edges and dense distribution of disease spots, where PMJDM can still completely identify multiple disease targets, while Faster-RCNN and YOLO11x miss some detections. This advantage is attributed to the candidate region generation module (N-RPN) in PMJDM that integrates HOG/LBP texture features, which effectively overcomes the mis-suppression of overlapping targets by traditional IoU thresholds through a sliding window strategy guided by local gradient information and an adaptive suppression mechanism based on texture similarity. Meanwhile, the multi-scale adaptive attention mechanism embedded in the shared feature extraction module significantly enhances the model’s sensitivity to small areas by dynamically fusing edge features at different scales.

The latter two sets of detection results further demonstrate PMJDM’s reliability advantage: in the third image set, PMJDM achieves the highest confidence score of 0.9 for the main disease spot area, outperforming Faster-RCNN (0.64) and YOLO11x (0.83) by 0.26 and 0.07, respectively. Moreover, the average confidence across the entire image improves significantly from 0.55/0.65 (comparison models) to 0.79. In the fourth set of images, the stable high confidence of PMJDM is equally prominent. This improvement can be attributed to three technical innovations: first, the post-processing module based on conditional random fields (CRF) effectively suppresses local misjudgments caused by uneven illumination through spatial continuity constraints and texture feature fusion; second, the dynamic weight adjustment mechanism ensures that the model maintains high-confidence decisions in complex scenes by balancing the gradient contributions of plant classification and disease detection tasks in real time; finally, the lightweight Transformer and CNN dual-branch structure significantly enhances the model’s representation ability for disease spot semantic information through cross-modal feature interaction. These technologies work synergistically, enabling PMJDM to achieve a dual breakthrough in detection accuracy and confidence while maintaining high inference speed, providing a reliable solution for precise disease diagnosis in complex agricultural environments.

### 4.3 Ablation experiment

**Table 5** illustrates the impact of removing individual PMJDM modules from its baseline mAP50 of 61.83%, revealing their collective importance. Without multi-scale attention, mAP50 drops to 59.41% (a 2.42% decrease), emphasizing its critical role in capturing features across object sizes, likely enhancing detection of small or large targets. Removing channel attention reduces mAP50 to 60.59% (1.24% drop), indicating its contribution to emphasizing key information by adjusting channel importance. Omitting texture filtering in N-RPN lowers mAP50 to 59.52% (2.31% drop), showing its importance in improving region proposal quality and detection accuracy.

**Table 5:** Ablation experiment results for PMJDM model.

Experiment Setting	Precision	Recall	mAP50	mAP50-95
Full PMJDM	71.84%	61.96%	61.83%	42.69%
Exp-1 (w/o Multi-Scale Attention)	70.13%	60.27%	59.41%	38.92%
Exp-2 (w/o Channel Attention)	70.68%	57.83%	60.59%	41.62%
Exp-3 (w/o Texture Filtering in N-RPN)	70.91%	60.42%	59.52%	41.38%
Exp-4 (w/o CRF Post-Processing)	70.29%	61.03%	60.97%	41.57%
Exp-5 (w/o Dynamic Weight Adjustment)	70.47%	60.61%	60.38%	41.46%
Exp-6 (w/o Deformable Convolution)	70.19%	60.08%	58.97%	41.06%
Exp-7 (w/o Soft NMS)	71.02%	60.39%	60.74%	41.83%

Excluding CRF post-processing results in an mAP50 of 60.97% (0.86% drop), a smaller decline suggesting it refines bounding boxes and reduces false positives, though it’s not the primary contributor.

Removing dynamic weight adjustment decreases mAP50 to 60.38% (1.45% drop), underlining its role in adaptively balancing module contributions for stability. Eliminating deformable convolution cuts mAP50 to 58.97% (2.86% drop)—the largest decline—proving its essential role in handling deformations and complex backgrounds. Dropping Soft NMS reduces mAP50 to 60.74% (1.09% drop), indicating its advantage over traditional NMS in managing overlapping boxes. As shown in **Table 5**, these modules—particularly deformable convolution and multi-scale attention—collectively drive PMJDM’s efficient detection capability, validating its design effectiveness.

**Table 6** demonstrates how varying hyperparameter settings affects PMJDM’s performance, with optimized values yielding the best outcomes. With anchor number K=9, mAP50 reaches 61.83%; reducing to K=3 drops it to 59.92% (1.91% lower) due to insufficient anchor coverage increasing misses, while K=12 yields 61.37% (0.46% lower) as redundancy reduces efficiency, making K=9 optimal. Anchor base size Base=8 achieves 61.83% mAP50; Base=4 falls to 60.33% (1.5% drop) due to poor large-object localization, and Base=16 drops to 60.62% (1.21% lower) from weaker small-object matching, confirming Base=8 as the best fit.

**Table 6:** Ablation experiment results for hyperparameters in PMJDM model.

Experiment Setting	Precision	Recall	mAP50	mAP50-95
Anchor Number (K)				
Baseline (K=9)	71.84%	61.96%	61.83%	42.69%
Exp-1 (K=3)	70.51%	60.28%	59.92%	41.13%
Exp-2 (K=6)	71.03%	61.17%	60.89%	41.94%
Exp-3 (K=12)	71.29%	61.42%	61.37%	42.25%
Anchor Base Size				
Baseline (Base=8)	71.84%	61.96%	61.83%	42.69%
Exp-4 (Base=4)	70.59%	60.47%	60.33%	41.52%
Exp-5 (Base=16)	70.88%	60.71%	60.62%	41.67%
Scales & Aspect Ratios				
Baseline (Scales={8, 16, 32}, Ratios={0.5, 1, 2})	71.84%	61.96%	61.83%	42.69%
Exp-6 (Scales={16}, Ratios={1})	70.42%	60.03%	59.71%	40.98%
Exp-7 (Scales={4, 8, 16, 32}, Ratios={0.33, 0.5, 1, 2, 3})	71.27%	61.38%	61.29%	42.14%
Learning Rate				
Baseline (LR=0.002→0.00002)	71.84%	61.96%	61.83%	42.69%
Exp-8 (LR=0.001→0.00001)	70.63%	60.49%	60.38%	41.42%
Exp-9 (LR=0.005→0.00005)	70.87%	61.12%	60.95%	41.89%
Batch Size				
Baseline (BS=16)	71.84%	61.96%	61.83%	42.69%
Exp-10 (BS=8)	71.06%	61.24%	61.05%	42.11%
Exp-11 (BS=32)	71.32%	61.49%	61.38%	42.34%

The scale and ratio configuration Scales={8,16,32}, Ratios={0.5,1,2} delivers 61.83% mAP50; simplifying to Scales={16}, Ratios={1} reduces it to 59.71% (2.12% drop) due to limited diversity, while expanding to Scales={4,8,16,32}, Ratios={0.33,0.5,1,2,3} yields 61.29% (0.54% lower) as added complexity lowers efficiency, affirming the original setup as ideal. A learning rate decaying from 0.01 to 0.0001 secures 61.83% mAP50; 0.05 to 0.005 drops to 60.38% (1.45% lower) due to initial instability, and 0.005 to 0.00005 falls to 60.95% (0.88% drop) from insufficient convergence, proving the original schedule optimal. Batch size BS=16 achieves 61.83% mAP50; BS=8 drops to 61.05% (0.78% lower) due to reduced stability, and BS=32 yields 61.38% (0.45% lower) from memory strain, making BS=16 the best compromise. As depicted in **Table 6**, these tuned hyperparameters ensure PMJDM’s training efficiency and exceptional performance.

## 5 Discussion

The experimental results underscore the superior performance of the PMJDM model across multiple evaluation metrics, including precision, recall, mAP50, and mAP50-95, when compared to established models like Faster-RCNN, Mask R-CNN, and various YOLO iterations. With a precision of 71.84% and recall of 61.96%, PMJDM demonstrates a marked improvement over Faster-RCNN (59.54% precision, 52.65% recall) and YOLOv10x (67.53% precision, 57.37% recall), reflecting its enhanced ability to accurately detect and localize plant diseases while minimizing false positives and negatives. Ablation studies further reveal the pivotal contributions of key components such as the multi-scale attention mechanism, deformable convolution, and dynamic weight adjustment, with their removal causing significant drops in mAP50 (e.g., 2.86% without deformable convolution). Visualization of detection results highlights PMJDM’s robustness in challenging scenarios, such as dense disease distributions and blurred leaf edges, attributing this success to the integration of texture-based candidate region generation and conditional random field post-processing, which ensure high-confidence predictions and spatial consistency. These findings suggest that PMJDM’s synergistic design not only elevates detection accuracy but also maintains computational efficiency (145.76 GFLOPs, 113 FPS), making it a highly effective solution for precision agriculture.

PMJDM’s robustness under varying lighting conditions is enhanced by the CRF post-processing module, which uses spatial constraints and texture feature fusion to reduce misclassifications from uneven illumination, achieving a confidence score of 0.79 in complex scenes, surpassing YOLO11x’s 0.65. However, performance in extreme lighting, such as strong backlighting, may be limited due to insufficient dataset coverage. Other limitations include PMJDM’s restriction to 5 plant species and 28 disease categories, requiring retraining for new classes, and its 49.1M parameters, which challenge ultralow-power device deployment. Future work could address these through expanded datasets and model compression. Additionally, PMJDM does not directly predict disease severity, though confidence scores indirectly reflect disease prominence. Incorporating severity annotations and regression models could enhance its utility. These improvements would make PMJDM more adaptable to diverse agricultural scenarios, strengthening its value in precision agriculture.

## 6 Conclusion

With global food demand rising rapidly, manual plant disease detection is slow and error-prone, while automated methods falter in complex scenes. The PlantDisease Multi-task Joint Detection Model (PMJDM), integrating enhanced ConvNeXt feature extraction, HOG/LBP-augmented N-RPN, multi-task branches, and CRF post-processing, offers an efficient solution. On a 26,073-image dataset, PMJDM achieves 71.84% precision, 61.96% recall, and 61.83% mAP50, outperforming Faster-RCNN (51.49%) and YOLOv10x (59.52%). PMJDM reduces redundancy via joint inference, balances tasks with dynamic weights, and enhances lighting robustness with CRF post-processing. Experiments show a 0.79 confidence in blurred leaf scenarios, surpassing YOLO11x’s 0.65. With 49.1M parameters and 113 FPS, PMJDM enables realtime use, aiding pesticide reduction and yield improvement. Future work may incorporate light-adaptive preprocessing, model compression, and severity prediction, expanding to more plant types and extreme conditions, advancing smart farming.

## Data Availability

The raw data supporting the conclusions of this article will be made available by the authors, without undue reservation.
